# Association between the serum lipoprotein-associated phospholipase A_2_ level and acute coronary syndrome

**DOI:** 10.5830/CVJA-2022-056

**Published:** 2023-01-11

**Authors:** Le Dong, Jiayi Tong, Shimin Fan

**Affiliations:** Department of Cardiovascular Medicine, Jiangbei Campus, Zhongda Hospital affiliated to Southeast University, Nanjing, Jiangsu Province, China

**Keywords:** acute coronary syndrome, lipoprotein-associated phospholipase A2, biochemical index, angiography

## Abstract

**Aim:**

The aim of this study was to explore the association between serum lipoprotein-associated phospholipase A2 (Lp-PLA2) level and acute coronary syndrome (ACS), and to analyse the correlations of Lp-PLA2 concentration with highsensitivity C-reactive protein (hs-CRP) level, body mass index (BMI), triglyceride (TG), troponin I (TNI), low-density lipoprotein cholesterol (LDL-C) and high-density lipoprotein cholesterol (HDL-C) levels, and severity of coronary artery disease.

**Methods:**

A total of 75 patients were divided into an unstable angina (UA) group (n = 54) and an acute myocardial infarction (AMI) group (n = 21). Another 72 subjects with normal coronary angiography results were selected as a control group. The levels of hs-CRP, TG, LDL-C, HDL-C and TNI were determined. Serum Lp-PLA2 concentration was measured by enhanced immunoturbidimetry. The Gensini score was obtained based on coronary angiography results.

**Results:**

The serum Lp-PLA2 concentration significantly increased in the AMI and UA groups compared with that of the control group (p < 0.05). Compared with the UA group, the AMI group had significantly increased levels of hs-CRP and TNI and higher Gensini score (p < 0.05). The UA group had increased levels of hs-CRP and higher Gensini score compared with those of the control group (p < 0.05), while the two groups had similar TNI levels (p > 0.05). Using serum Lp-PLA2 concentration as the dependent variable, and hs-CRP, TG, LDL-C, HDL-C, TNI and Gensini score as independent variables, the analysis results showed that Lp-PLA2 concentration was positively correlated with BMI and hs-CRP, LDL-C and TNI levels.

**Conclusions:**

There was a positive correlation between Lp-PLA2 concentration and LDL-C level, therefore plasma LDL-C level should be controlled to prevent ACS.

Due to coronary atherosclerosis and vascular plaque instability, plaque rupture and erosion occur under a series of triggers, thus causing coronary thrombosis and incomplete or complete occlusion of the corresponding blood vessels, and ultimately, resulting in acute myocardial ischaemia and hypoxia. Whether ACS occurs is primarily dependent on the plaque stability, and has no direct relationship with the plaque size.^1^

With the deepening of research, there has been considerable evidence that the aggregation of inflammatory cells and the release of inflammatory markers are the main culprits throughout the development process of atherosclerotic plaque. At present, it is well accepted that coronary atherosclerosis is a kind of chronic vascular inflammation,^2^ and the hypothesis that ‘inflammation causes cardiovascular events’ has become a research hotspot. The associations of some inflammatory factors, such as highsensitivity C-reactive protein (hs-CRP), CRP, interleukin-37 (IL- 37) and IL-38, with the onset and prognosis of coronary heart disease have been revealed.^3^

Lipoprotein-associated phospholipase A2 (Lp-PLA2), a newly discovered inflammatory marker, plays an important role in the occurrence and development of atherosclerosis and the rupture of unstable plaques. The rupture of unstable plaques is crucial to the occurrence of ACS.

Lp-PLA2, as an inflammatory marker, can serve as a predictor for the risk of coronary heart disease events, and its elevation indicates a high risk for coronary heart disease.^4^,^5^ However, the associations of serum Lp-PLA2 concentration with hs-CRP level, body mass index (BMI), triglyceride (TG), troponin I (TNI), low-density lipoprotein cholesterol (LDL-C) and high-density lipoprotein cholesterol (HLD-C) levels, and the severity of coronary artery disease have seldom been reported.

In this article, the Lp-PLA2 concentration was compared between normal subjects and ACS patients to explore whether Lp-PLA2 could predict the occurrence of ACS. Moreover, the association between Lp-PLA2 and other clinical indicators was analysed, thereby contributing to early prevention and treatment of ACS.

## Methods

A total of 75 patients were enrolled and divided into an unstable angina (UA) group (n = 54) and an acute myocardial infarction (AMI) group (n = 21). Another 72 subjects with normal coronary angiography results were selected as a control group. This study was approved by the ethics committee of our hospital, and written informed consent was obtained from all patients.

The Lp-PLA2 kit and scattering turbidimetric analyser were purchased from Nanjing Norman Biological Technology Co, Ltd (China). Other apparatus included separation gel coagulationpromoting tubes, electric thermostatic oscillating water tank and ultra-high-speed centrifuge (Thermo Fisher Scientific, USA).

Venous blood (5 ml) was drawn from each patient in the AMI group immediately after admission, and fasting venous blood (5 ml) was drawn from each patient in the other groups in the morning, the day after admission. The blood samples were collected into separation gel coagulation-promoting tubes, gently turned upside down five to six times, and stood vertically for 30 minutes, followed by centrifugation at 3 000 r/min for 15 minutes. Then 15 μl of upper-layer serum was aspirated and stored at 4°C for tests within 48 hours.

Serum Lp-PLA2 concentration was measured by enhanced immunoturbidimetry using the Norman scattering turbidimetric analyser. In detail, reagent R1 [240 μl; 200 mmol/l pH 6.5 phosphate-buffered saline (PBS) and 1.30% PEG6000] and serum (12 μl) were well mixed. Then the mixture was added to reagent R2 (60 μl; 200 mmol/l pH 8.0 PBS and 0.2 g/l sensitised latex particles of mouse anti-human Lp-PLA2 monoclonal antibody) and immediately tested.

According to the American Heart Association, the severity of coronary artery stenosis was assessed using the Gensini scoring system.^6^ The coronary artery consists of left main coronary artery (LM), left anterior descending branch (LAD), left circumflex branch (LCX) and right coronary artery (RCA). There were no abnormal findings, and stenosis of ≤ 25, 26–50, 51–75, 76–90, 91–99 and 100% were recorded as 0, 1, 2, 4, 8, 16 and 32 points, respectively.

The diseased coronary artery was multiplied by the corresponding coefficient value: LM × 5, proximal LAD × 2.5, mid LAD × 1.5, distal LAD × 1, first diagonal branch × 1, second diagonal branch × 0.5, proximal LCX × 2.5, distal LCX and posterior descending branch × 1, posterior lateral branch × 0.5, proximal/mid/distal RCA and posterior descending branch × l. The score was calculated as follows: weight coefficient of coronary artery stenosis × weight coefficient of each diseased vessel, and the score of each branch was added up to obtain the total score.

## Statistical analysis

SPSS21.0 software (IBM Inc, USA) was used for statistical analysis. Measurement data are described as mean ± standard deviation, and count data are described as rate (%). The homogeneity test of variance was first conducted on measurement data, and the data were compared by the t-test among groups in the case of homogeneity of variance and by the rank sum test among groups in the case of heterogeneity of variance. Count data were compared by the χ2 test or Fisher’s exact probability test. Linear correlation analysis was performed for variables. A value of p < 0.05 was considered statistically significant.

## Results

No statistically significant differences were found in height, weight, BMI, and levels of TG, LDL-C and HDL-C in the UA, AMI and control groups (p > 0.05) (Table 1).

**Table 1:** General data (mean ± SD)

*Item*	*UA group (n = 72)*	*AMI group (n = 21)*	*Control group (n = 72)*
Height (m)	1.65 + 0.75	1.68 + 0.83	1.63 + 0.73
Body weight (kg)	68.80 + 10.21	71.14 + 8.38	68.61 + 9.57
BMI (kg/m²)	24.98 + 3.09	25.19 + 2.80	25.56 + 3.16
TG (mmol/l)	1.71 + 0.94	1.65 + 0.74	1.66 + 0.89
LDL-C (mmol/l)	2.67 + 0.76	2.78 + 0.89	2.68 + 0.62
HDL-C (mmol/l)	1.04 + 0.22	1.04 + 0.22	1.14 + 0.22

AMI: acute myocardial infarction; BMI: body mass index; HDL-C: high-density

lipoprotein cholesterol; LDL-C: low-density lipoprotein cholesterol; TG:

triglyceride; UA: unstable angina.

**Fig. 1:**
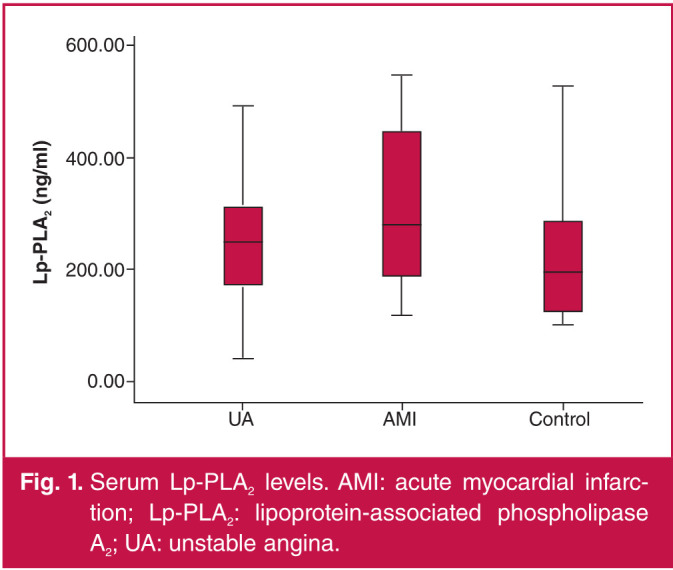
Serum Lp-PLA2 levels. AMI: acute myocardial infarction; Lp-PLA2: lipoprotein-associated phospholipase A2; UA: unstable angina.

The serum Lp-PLA2 concentrations significantly increased in both the AMI (313.81 ± 133.41 ng/ml) and UA groups (259.11 ± 121.45 ng/ml) compared with those in the control group (227.56 ± 118.84 ng/ml) (p < 0.05). There were no significant differences in the serum Lp-PLA2 concentrations between the UA and AMI groups (p > 0.05) (Fig. 1).

Compared with the UA group, the AMI group had significantly increased levels of hs-CRP (3.80, 2.50–15.35 vs 1.75, 1.10–3.32 mg/l) and TNI (1.94, 0.09–8.94 vs 0, 0 ng/ml), and increased Gensini score (46.00, 36.00–78.00 vs 18.00, 8.00– 36.50) (p = 0.00, 0.00, 0.00). The UA group had increased levels of hs-CRP (3.00–4.53 mg/l) and higher Gensini score (27.81– 33.81) compared with those of the control group (p = 0.04, 0.00), while the TNI level had no significant difference between the two groups (p > 0.05) (Table 2).

**Table 2:** Biochemical indices and Gensini scores

*Item*	*UA group (n = 72)*	*AMI group (n = 21)*	*Control group (n = 72)*
hs-CRP (mg/l)	1.75 (1.10-3.32)	3.80 (2.50-15.35)	2.70 (1.40-4.65)
TNI (ng/ml)	0	1.94 (0.09-8.94)	0
Gensini score	18.00 (8.00-36.50)	46.00 (36.00-78.00)	0

AMI: acute myocardial infarction; hs-CRP: high-sensitivity C-reactive protein;

TNI: troponin I; UA: unstable angina.

**Fig. 2:**
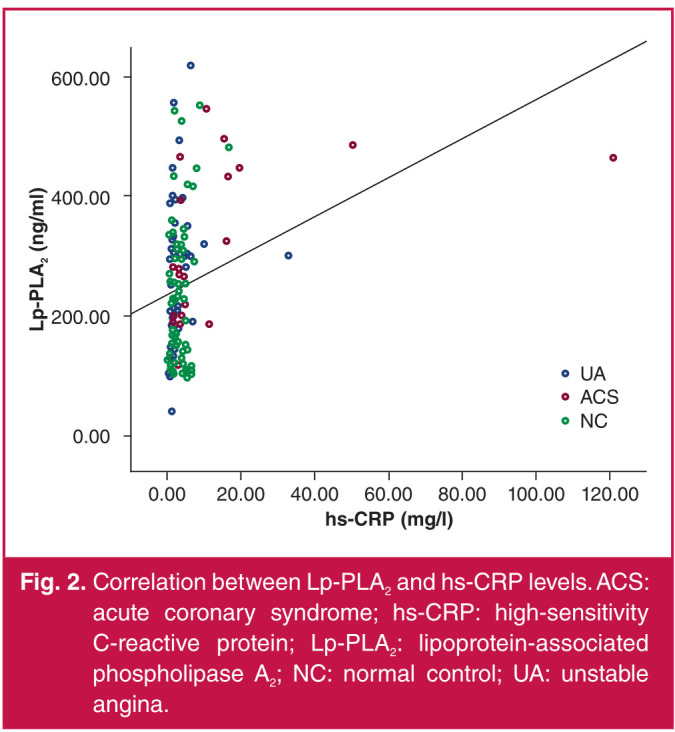
Correlation between Lp-PLA2 and hs-CRP levels. ACS: acute coronary syndrome; hs-CRP: high-sensitivity C-reactive protein; Lp-PLA2: lipoprotein-associated phospholipase A2; NC: normal control; UA: unstable angina.

**Fig. 3:**
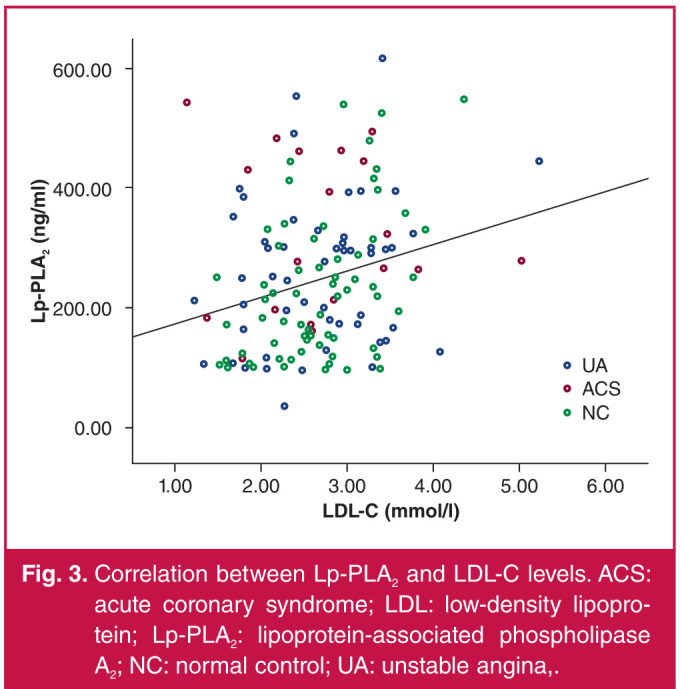
Correlation between Lp-PLA2 and LDL-C levels. ACS: acute coronary syndrome; LDL: low-density lipoprotein; Lp-PLA2: lipoprotein-associated phospholipase A2; NC: normal control; UA: unstable angina,.

**Fig. 4:**
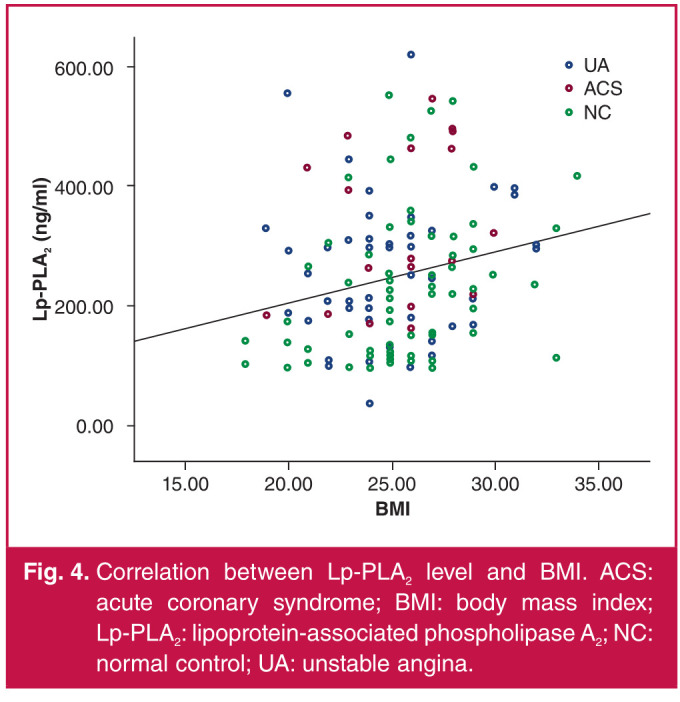
Correlation between Lp-PLA2 level and BMI. ACS: acute coronary syndrome; BMI: body mass index; Lp-PLA2: lipoprotein-associated phospholipase A2; NC: normal control; UA: unstable angina.

**Fig. 5:**
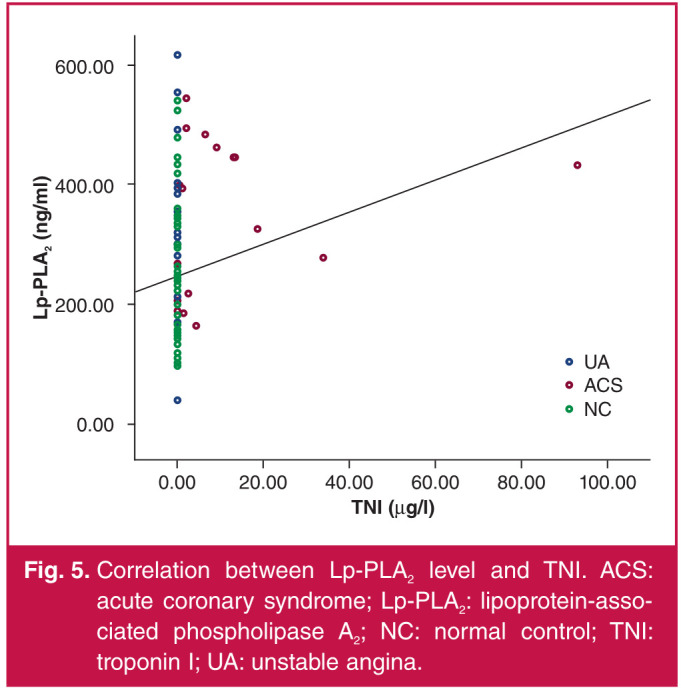
Correlation between Lp-PLA2 level and TNI. ACS: acute coronary syndrome; Lp-PLA2: lipoprotein-associated phospholipase A2; NC: normal control; TNI: troponin I; UA: unstable angina.

With serum Lp-PLA2 concentration as a dependent variable, and hs-CRP, TG, LDL-C, HDL-C, TNI and Gensini score as independent variables, the analysis results showed that Lp-PLA2 concentration was positively correlated with BMI and hs-CRP, LDL-C and TNI levels (p < 0.05) (Table 3, Figs 2–5).

**Table 3:** Correlations between Lp-PLA2 concentration and variables

*Independent variable*	*r*	*p-value*
Height (m)	0.06	0.47
Body weight (kg)	0.13	0.10
BMI (kg/m²)	0.21	0.01
hs-CRP (mg/l)	0.29	0
TG (mmol/l)	0.05	0.54
LDL-C (mmol/l)	0.25	0
HDL-C (mmol/l)	0.16	0.84
Gensini score	0.13	0.11
TNI (ng/ml)	0.18	0.02

BMI: body mass index; HDL-C: high-density lipoprotein cholesterol; hs-CRP: high-sensitivity C-reactive protein; LDL-C: low-density lipoprotein cholesterol; Lp-PLA2: lipoprotein-associated phospholipase A2; TG: triglyceride; TNI: troponin I.

## Discussion

As the most common cardiovascular disease in China currently, coronary heart disease, characterised by high mortality and disability rates, has brought great economic and psychological burden to families and society. ACS, one of the most common types of coronary heart disease in the clinic, has become the primary killer, threatening human health due to its high complication, disability and mortality rates. Its major pathophysiological mechanism is that due to the rupture of unstable plaques in coronary atherosclerosis and vasospasm induced by a variety of factors, platelet adhesion and aggregation, as well as secondary thrombosis occur.^7^ There is a close association between the formation of atherosclerotic plaques and ACS, and related cardiovascular events are caused by the rupture of unstable plaques.

Despite the certain significance of traditional risk factors (such as blood lipid levels) and cardiac imaging for the diagnosis and prevention of ACS, rupture of unstable plaque remains the direct cause of acute coronary events. However, the above methods fail to clearly reflect the rupture tendency of unstable plaques and identify the exact timing of rupture. Therefore, it has been a research hotspot in the cardiovascular field to search for biological indicators for plaque vulnerability, combine evidencebased medicine and individualised precision medicine in clinical practice, and actively apply the easy-to-detect biological markers to clinical patients.^8^

Lp-PLA2, a newly discovered cardiovascular-specific inflammatory mediator, is closely related to the occurrence and development of cardiovascular events, which can facilitate the formation of atherosclerotic plaques and lead to the rupture of unstable plaques. Early examination has not only positive clinical significance for early detection of possible cardiovascular events, but also profound significance for easing the economic burden of patients and even reducing the national medical investment.^9^ A high correlation between the occurrence of ACS and Lp-PLA2 concentration has been found. By early monitoring of Lp-PLA2 concentration and active intervention, ACS can be prevented.^10^ Discovered in 1980, Lp-PLA2 is a platelet-activating factor acetyl hydrolase, synthesised and secreted by mature macrophages and lymphocytes, which can promote the hydrolysis of glycerophospholipid acyl lipid bond and lipoproteins on the cell membrane surface, ultimately generating lysophospholipase and non-esterified fatty acids. Its secretion is regulated by inflammatory mediators. After entering the blood circulation, 80% of Lp-PLA2 will bind to LDL-C to hydrolyse oxidised lecithin and produce pro-inflammatory substances. Therefore, Lp-PLA2 can contribute to inflammation and atherosclerosis.^11^

As pointed out in an article in the Lancet by Zhao et al.,^12^ increased levels of serum Lp-PLA2 were closely associated with the risk of ACS. Mourouzis et al.^13^ found a close correlation between Lp-PLA2 concentration and the prognosis of ACS. Wu et al.^14^ showed that ACS patients had a significantly higher level of serum Lp-PLA2 (233 ± 54 μg/l) than patients without coronary artery disease (208 ± 52 μg/l), with a statistically significant difference (p < 0.01), and the Lp-PLA2 concentration was correlated with the change in LDL-C level. As proven by Jabor et al.,^15^ lipid-lowering therapy lowered the serum Lp-PLA2 level while reducing the serum LDL-C level, also suggesting the correlation between Lp-PLA2 and LDL-C. Yang et al.^16^ also found that Lp-PLA2 concentration was positively correlated with the occurrence of ACS.

In this study, the Lp-PLA2 concentration was significantly higher in the AMI and UA groups than that in the control group, and the difference was statistically significant. This means the plasma Lp-PLA2 concentration was significantly higher in ACS, with a statistically significant difference. Moreover, the serum Lp-PLA2 concentration was positively correlated with LDL-C level. The results of this study were consistent with those obtained by the above scholars, namely that Lp-PLA2 is involved in the inflammatory response in ACS, leading to the progression of unstable plaques. It can therefore be used as a vascular inflammatory factor to monitor the occurrence of ACS, and ACS can be prevented by controlling plasma LDL-C levels.

Ma et al.^17^ pointed out that Lp-PLA2 concentration had no statistical correlation with the Gensini score, consistent with our findings that Lp-PLA2 concentration had no correlation with the severity of coronary artery stenosis. Besides, the serum Lp-PLA2 concentration was related to the BMI, suggesting that weight control may be beneficial to preventing ACS, which needs further study.

Significant results were obtained in this study, but further research is still needed to make up for the following deficiencies. Lp-PLA2 could be used as an inflammatory marker to predict the occurrence of ACS, but the expected results were not obtained in the subgroup comparison and analysis, so further randomised clinical trials are needed. There was no correlation between Lp-PLA2 level and the severity of coronary artery stenosis, but the relationship between Lp-PLA2 concentration and stable/ unstable plaques is not described, as it is pending further research.

## Conclusion

The Lp-PLA2 concentration was significantly higher in the AMI and UA patients than that in subjects with normal coronary angiography results, and it was positively correlated with hs-CRP levels, suggesting that Lp-PLA2 is probably an inflammatory factor related to ACS. There was a positive correlation between Lp-PLA2 concentration and LDL-C level, so it is of significance to control plasma LDL-C levels to prevent ACS. Lp-PLA2 concentration had no correlation with the Gensini score, indicating that it has no correlation with the severity of coronary artery disease.
